# Supplementary material for: Age, Disease Severity and Ethnicity Influence Humoral Responses in a Multi-Ethnic COVID-19 Cohort
Source: Viruses. 2021 Apr 28;13(5):786. doi: 10.3390/v13050786 (PMC8146997; doi:10.3390/v13050786)
Supplement: Supplementary file 1 [file viruses-13-00786-s001.zip › viruses-1178397-supplementary.pdf]

Supplementary information

Age, disease severity and ethnicity influence humoral responses in a multi-ethnic COVID-19 cohort

Muneerah Smith[1‡], Houari B. Abdesselem[2,3‡], Michelle Mullins[1‡], Ti-Myen Tan[4‡], Andrew J.M. Nel[1], Maryam A.Y. Al-Nesf[5], Ilham Bensmail[2], Nour K. Majbour[2], Nishant N. Vaikath[2], Adviti Naik[2], Khalid Ouararhni[2], Vidya Mohamed-Ali[6], Mohammed Al-Maadheed[6], Darien T. Schell[1], Seanantha Baros[1], Nur D. Anuar[4], Nur H. Ismail[4], Priscilla E. Morris[4], Raja N.R. Mamat[4], Nurul S.M. Rosli[4], Arif Anwar[4], Kavithambigai Ellan[7], Rozainanee M. Zain[7], Wendy A. Burgers[8,9], Elizabeth S. Mayne[10], Omar M.A. El-Agnaf[2*], Jonathan M. Blackburn[1,4,9*]

[1] Department of Integrative Biomedical Sciences, Faculty of Health Sciences, University of Cape Town, South Africa.
[2] Neurological Disorders Research Center, Qatar Biomedical Research Institute (QBRI), Hamad Bin Khalifa University, Qatar. Foundation, Doha, Qatar.
[3] Proteomics Core Facility, Qatar Biomedical Research Institute (QBRI), Hamad Bin Khalifa University, Qatar Foundation, Doha, Qatar.
[4] Sengenics Corporation, Level M, Plaza Zurich, Damansara Heights, Kuala Lumpur 50490, Malaysia.
[5] Hamad General Hospital, Hamad Medical Corporation, Doha, Qatar.
[6] Anti-Doping Laboratory Qatar, Doha, Qatar.
[7] Institute for Medical Research, Virology Lab, Level 2, Block C7, Infectious Disease Research Centre, Setia Alam, Selangor 40170, Malaysia.
[8] Division of Medical Virology, Department of Pathology, University of Cape Town, Cape Town, South Africa; and Wellcome Centre for Infectious Diseases Research in Africa, University of Cape Town, Cape Town, South Africa.
[9] Institute of Infectious Disease and Molecular Medicine, Faculty of Health Sciences, University of Cape Town, South Africa.
[10] Department of Immunology, National Health Laboratory Service (NHLS) and University of the Witwatersrand, Charlotte Maxeke Johannesburg Academic Hospital, Johannesburg, South Africa.
‡ These authors contributed equally to the work
* Corresponding author: jonathan.blackburn@uct.ac.za; Tel: +27 21 406 6071
* Corresponding author: oelagnaf@hbku.edu.qa; Tel: +974 5593 5568

Supplementary Tables

Table S1. Oligo pairs used to construct the N-core, NTD, and CTD clones.

Construct	Primer	Oligo sequence
N-core	**Forward**	**5'- ATT ATC TAT AAC TAG TAT GGG ACT GCC TAA CAA CAC CGC TTC ATG G -3'**
	**Reverse**	**5'- ATT TCG CAT CCA TGG GGA GCC GCC CCC TCC AGT CTT GTA GGC GTC GAT GTG CTT GTT CAG CAG G -3'**
NTD	**Forward**	**5'- ATT ATC TAT AAC TAG TAT GCA GGG ACT GCC TAA CAA CAC CGC TTC ATG G -3**
	**Reverse**	**5'- ATT TCG CAT CCA TGG GGA GCC GCC CCC TCC ACC ACC ACG GGA ACC CTC AGC G -3'**
CTD	**Forward**	**5'- ATT ATC TAT AAC TAG TAT GGT GAC CAA GAA GTC CGC TGC TGA GGC C -3'**
	**Reverse**	**5'- ATT TCG CAT CCA TGG GGA GCC GCC CCC TCC GAA AGT CTT GTA GGC GTC GAT GTG CTT GTT CAG C -3'**

Table S2. Amino acid sequences and locations of the 17 tiling peptides for the detection of SARS-CoV-2 nucleocapsid phosphoprotein. Location in protein is based on SARS-CoV-2 nucleocapsid protein (Genbank accession number YP_009724397.2). (Ac) refers to Acetyl. All peptides listed have a N-terminal -(PEG)6-CH2-CH2-CONH-K(Bio) modification.

Peptide	Peptide	Length	Starting Position	Ending Position
**1/ IDR1**	APRITFGGPSDSTGSNQN	18	12	29
**2**	DFSKQLQQSMSSADS	15	402	416
**3**	EAGLPYGANKDGIIWVA	17	118	134
**4**	EPKKDKKKKADETQAL	16	367	382
**5**	ERSGARSKQRRPQGL	15	31	45
**6/IDR6**	LLPAADLDDFSKQLQQSM	18	394	411
**7**	LQQSMSSADSTQA	13	407	421
**8/IDR8**	MAGNGGDAALALLLLDRL	18	210	227
**9**	PSDSTGSNQNGERSGARSKQRRPQ	24	20	43
**10**	QRQKKQQTVTLLPAADLDDFSKQ	23	384	406
**11**	Q-NQRNAPRITFGGPSDS	18	6	23
**12**	RITFGGPSDSTGSNQNGER	19	14	32
**13**	SRGTSPARMAGNGGDAAL	18	202	219
**14**	STGSNQNGERSGARSKQR	18	23	40
**15**	TGSNQNGERSGARSKQRRP	19	24	42
**16/IDR16**	T(Ac)EPKKDKKKKADETQALPQRQKK	23	366	388
**17**	TEPKKDKKKKADETQALPQRQKK	23	366	388

Table S3. Microarray assay sensitivity as a function of time post onset of symptoms, and further separated as a function of disease severity.

Days post symptom onset	Number of patients	Positive on immunoassay	Sensitivity (%)
**0-7**	24	15	62.5
**8-14**	61	49	67.5
**15-21**	12	9	75.0
**22+**	3	2	66.7
**Mild**			
**0-7**	16	9	56.3
**8-14**	27	16	59.3
**15-21**	5	3	60.0
**22+**	2	1	50.0
**Severe**			
**0-7**	8	6	75.0
**8-14**	34	33	97.1
**15-21**	7	6	85.7
**22+**	1	1	100

Table S4. Positive and negative predictor values in Cohort 3

Disease severity	Positive/Negative	RT-PCR status		PPV	NPV
		True positives/negatives	Total positive/negative tests		
**All samples (Case, n=100; Control, n=38)**	Positive	75	79	0.95	-
	Negative	34	59	-	0.58
**Mild (Case, n=50; Control, n=38)**	Positive	29	33	0.88	-
	Negative	34	55	-	0.62
**Severe (Case, n=50, Control, n=38)**	Positive	46	50	0.92	-
	Negative	34	38		0.89

Table S5: Percentage of each ethnicity in Cohort 3, compared to the percentage of each ethnicity found in the Qatari cohort. Ethnicities that did not fall under the three broader ethnic groups were excluded from this table (n=5)

Ethnic group	Ethnicity	Number of individuals in cohort	Percentage of cohort (%)	Percentage of population (%)
**Middle Eastern (other)**	Egyptian	5	5	9.35
	Iranian	1	1	1
	Sudanese	2	2	1.9
	Syrian	1	1	1.7
	Yemeni	1	1	Not reported
	Jordanian	0	0	1.6
	Lebanese	0	0	1.25
	Tunisian	0	0	0.8
	Turkish	0	0	0.3
	Saudi Arabian	0	0	0.25
	Iraqi	0	0	0.2
	**Total**	**10**	**10**	**18.35**
**Middle Eastern (Qatari)**	Qatari	**15**	**15**	**10.5**
**South Asian**	Bangladeshi	18	18	12.5
	Filipino	7	7	7.35
	Indian	24	24	21.8
	Maylasian	0	0	0.15
	Nepalese	18	18	12.5
	Pakistani	2	2	4.7
	Sri-lankan	1	1	4.35
	Afghan	0	0	0.12
	Indonesian	0	0	0.85
	**Total**	**70**	**70**	**64.32**

Supplementary Figures

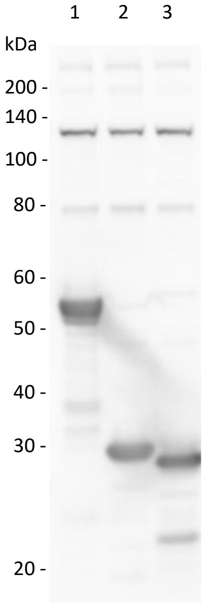

Figure S1. Western blot of SARS-2-nucleocapsid structural domains. Total proteins of lysed clarified *S. frugiperda* cells were analysed on a NuPAGE™ 4 to 12%, Bis-Tris (Thermo) SDS-PAGE gel. Subsequent western blot was analysed using streptavidin-horseradish peroxidase conjugate monoclonal antibodies (Cytiva). Lanes 1 N-core, 2 C-terminal domain (CTD), 3 N- terminal domain (NTD).

A

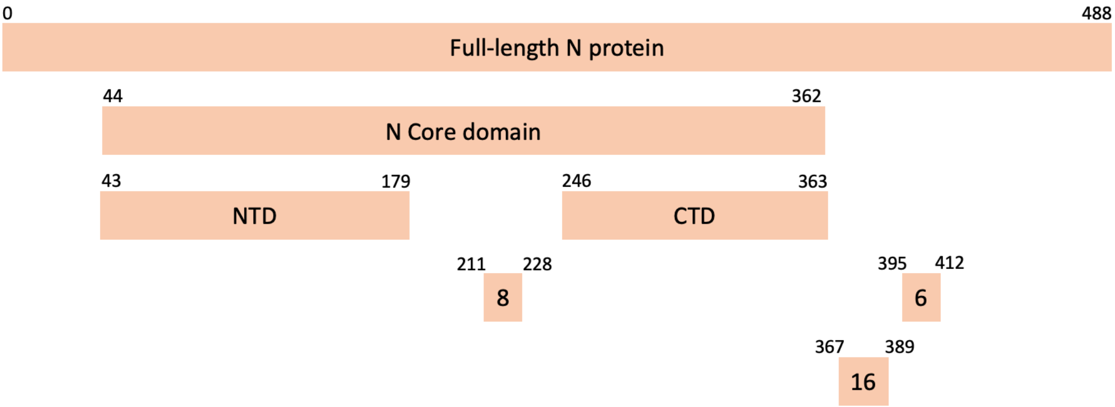

B

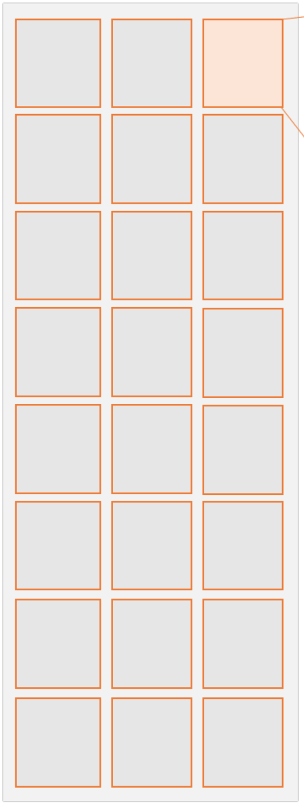

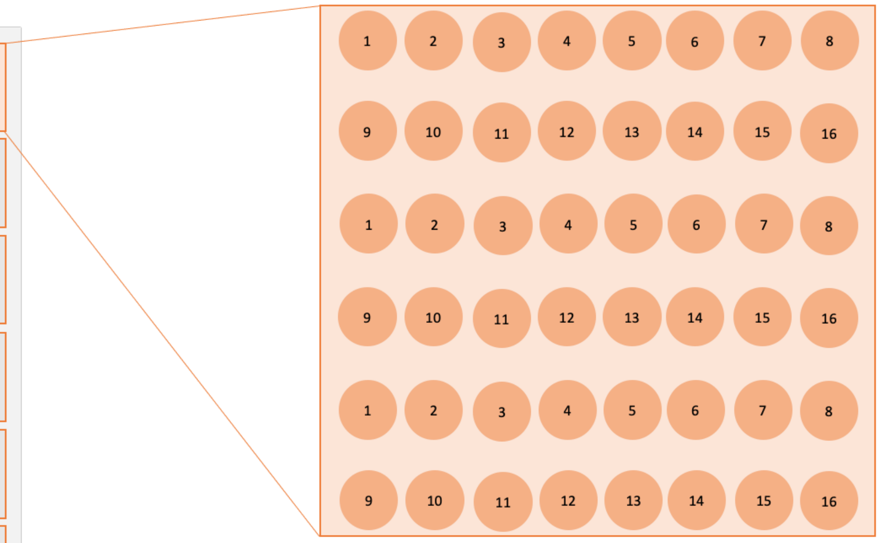

1	Cy3-bBSA	9	SARS-CoV-2 S RBD
2	IgM	10	SARS-CoV-2 S Ectodomain trimer
3	IgM	11	SARS-CoV-2 N IDR Epitope 16
4	IgG	12	SARS-CoV-2 N IDR Epitope 8
5	IgG	13	SARS-CoV-2 N IDR Epitope 6
6	IgA	14	SARS-CoV-2 N NTD
7	IgA	15	SARS-CoV-2 N CTD
8	SARS-CoV-2 S1	16	SARS-CoV-2 N Core domains

Figure S2. SARS-CoV-2 N protein amino acid coverage on SARS-CoV-2 microarray. (A) Final N protein constructs fabricated on the SARS-CoV-2 microarray, including the full length protein, core domain (amino acids 44-362), N-terminal domain (NTD) (amino acids 43-179), C-terminal domain (CTD) (amino acids 246-363), peptide 6 (amino acids 395-412), peptide 8 (amino acids 211-228) and peptide 16 (amino acids 367-389). (B) Final microarray layout: 24-plex array with 16 probes (summarised in the Table insert) printed in triplicate.

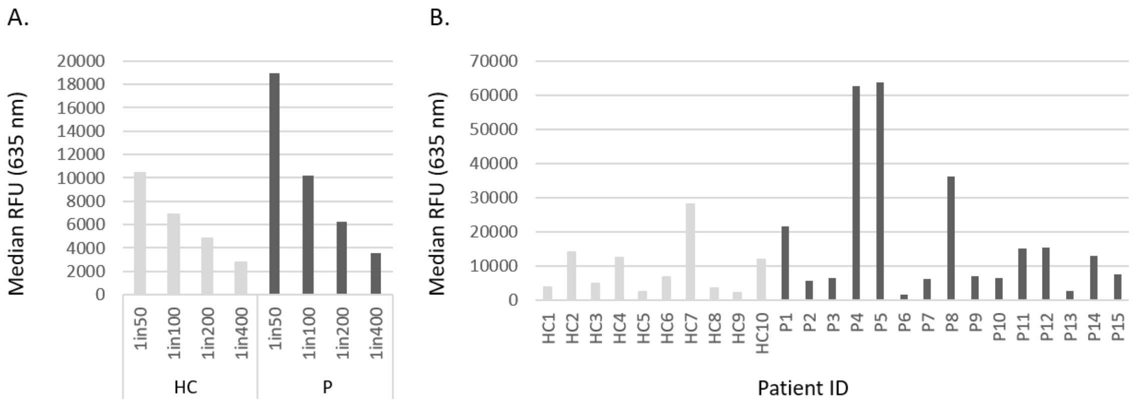

Figure S3. IgG response to SARS-CoV-2 full-length N protein. The IgG response of pooled healthy controls (HCs) and convalescent COVID-19 patients (Ps) was assessed against the full-length recombinant SARS-CoV-2 N protein. (A) A serial dilution (1:50, 1:100, 1:200 and 1:400) of pooled healthy controls (HCs) and convalescent COVID-19 patients (Ps) was tested against recombinant full-length SARS-CoV-2 N protein to determine specificity and the optimal serum/plasma dilution. (B) The IGG response of individual HCs and Ps were also assayed to determine the frequency of non-specific binding in HCs.

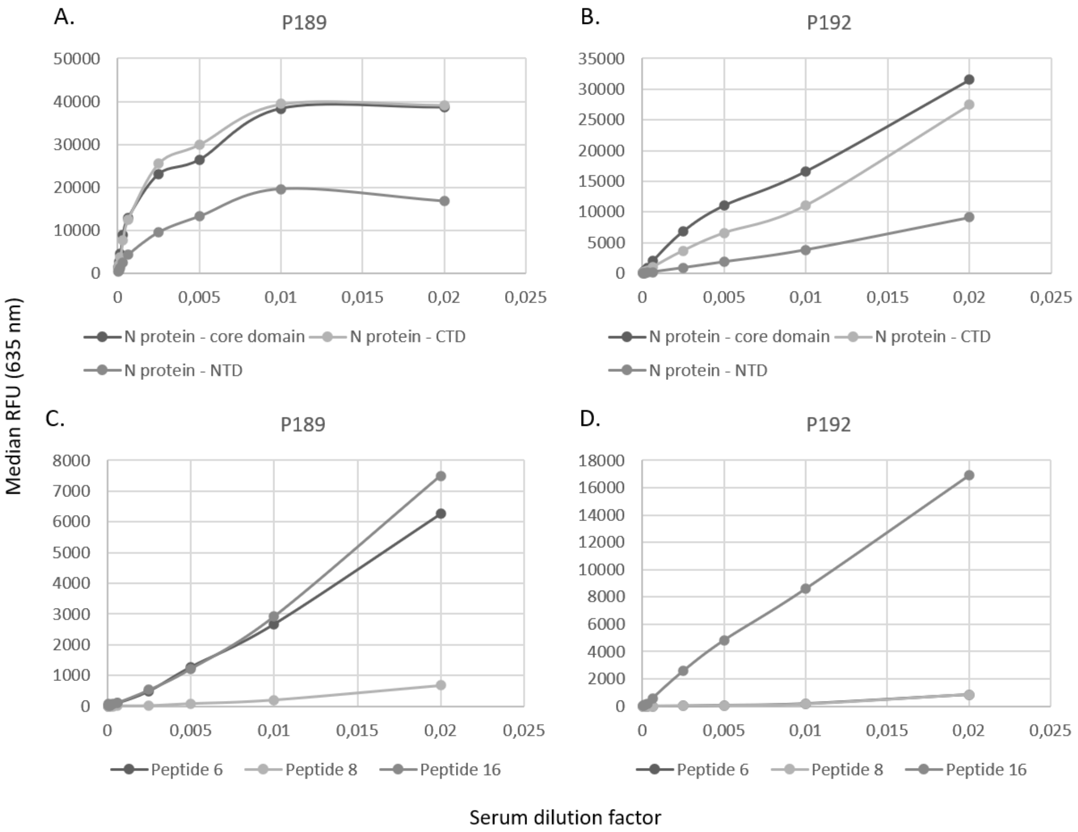

Figure S4. Linearity of signal as a function of serum dilution on the microarray platform. Panels A & B show data for the N core domains, N-terminal domain and C-terminal domain, for patients 189 and 192 respectively. Panels C & D show data for peptides 6, 8 & 16, for patients 189 and 192 respectively. x-axis shows the serum dilutions for each measurement. y-axis units are relative fluorescence units (RFU).

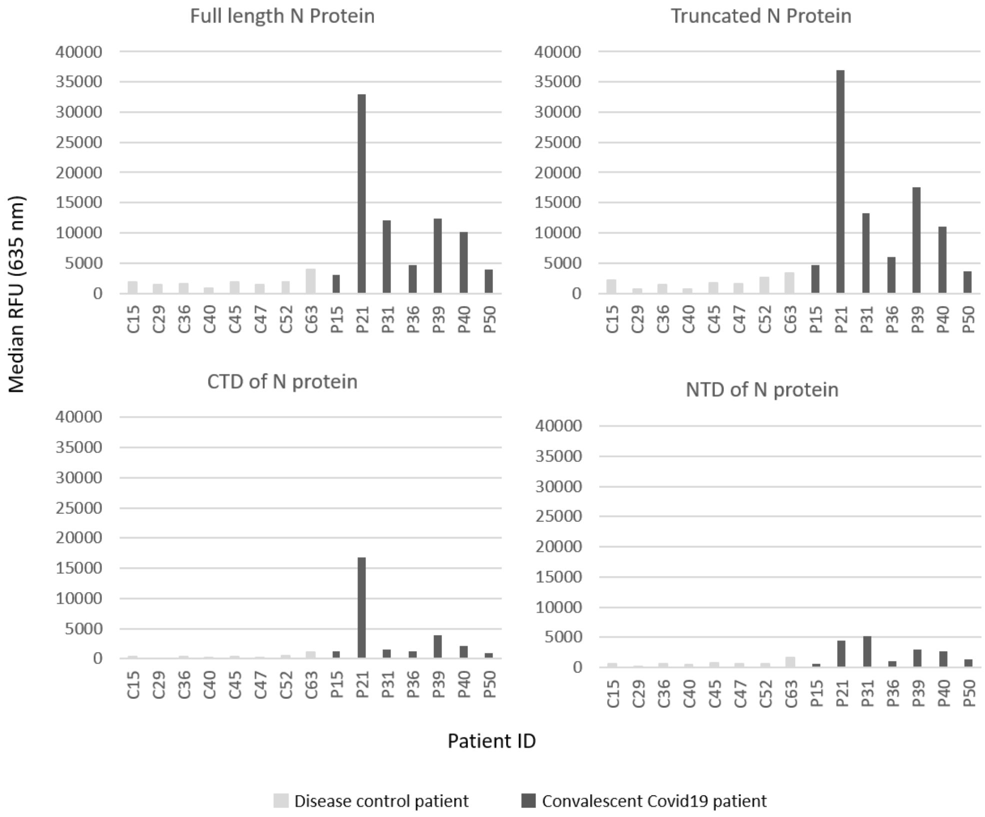

Figure S5. IgG responses to SARS-CoV-2 N protein variants. Four SARS-CoV-2 N protein variants were fabricated on to the microarray surface, including full-length N protein, core domain (amino acids 44-364), C-terminal domain (CTD) (amino acids 248-365) and N terminal domain (NTD) (amino acids 24-181). The IgG response from the plasma of 8 colorectal cancer (C) and 7 convalescent COVID-19 (P) patients were assessed for the 4 variants.

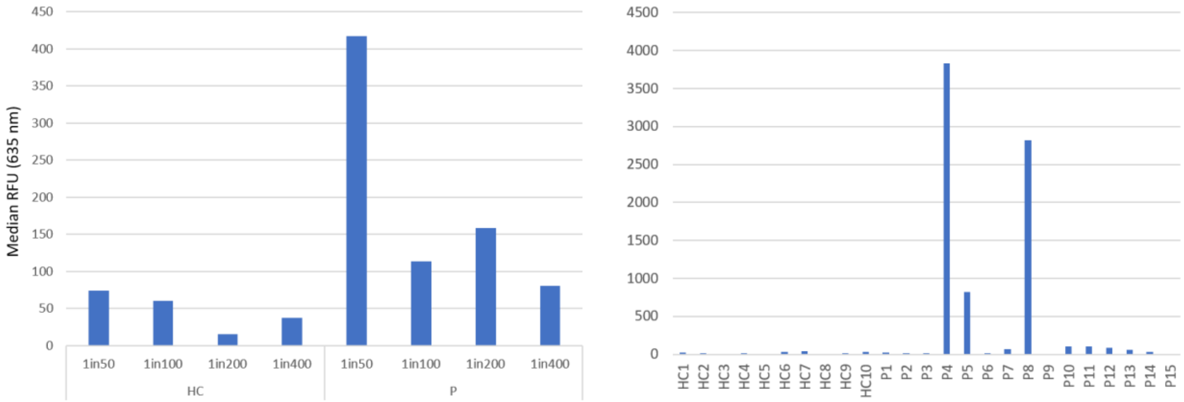

Figure S6. Antibody response to peptide 1. The IgG response of pooled healthy controls (HCs) and convalescent COVID-19 patients (Ps) was assessed against peptide 1. (A) A serial dilution (1:50, 1:100, 1:200 and 1:400) of pooled healthy controls (HCs) and convalescent COVID-19 patients (Ps) was tested against recombinant full-length SARS-CoV-2 N protein to determine specificity and the optimal serum/plasma dilution. (B) The IgG response of individual HCs and Ps were also assayed to determine the frequency of non-specific binding in HCs.

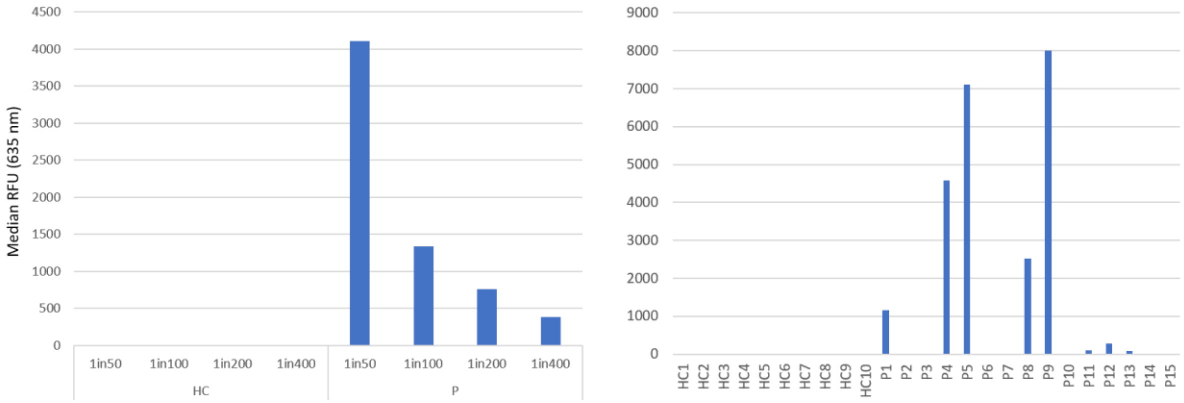

Figure S7. Antibody response to peptide 2. The IgG response of pooled healthy controls (HCs) and convalescent COVID-19 patients (Ps) was assessed against peptide 2. (A) A serial dilution (1:50, 1:100, 1:200 and 1:400) of pooled healthy controls (HCs) and convalescent COVID-19 patients (Ps) was tested against recombinant full-length SARS-CoV-2 N protein to determine specificity and the optimal serum/plasma dilution. (B) The IgG response of individual HCs and Ps were also assayed to determine the frequency of non-specific binding in HCs.

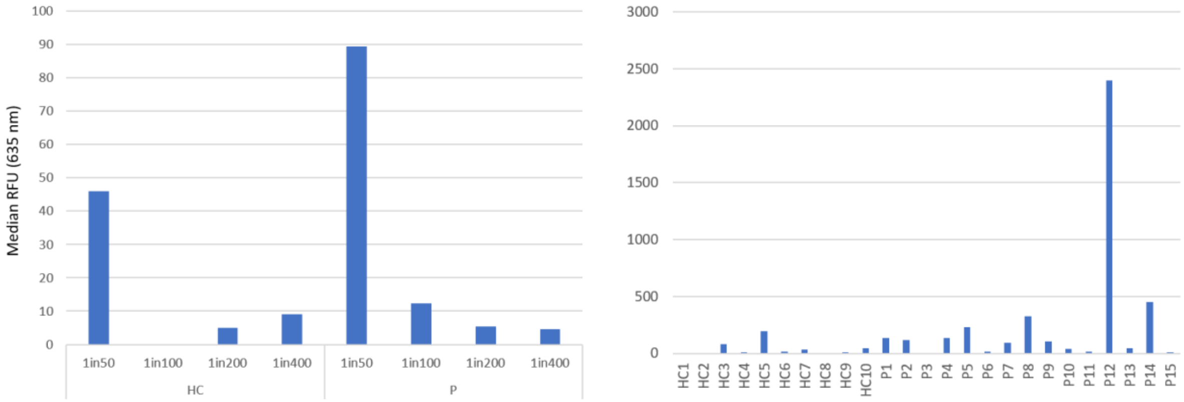

Figure S8. Antibody response to peptide 3. The IgG response of pooled healthy controls (HCs) and convalescent COVID-19 patients (Ps) was assessed against peptide 3. (A) A serial dilution (1:50, 1:100, 1:200 and 1:400) of pooled healthy controls (HCs) and convalescent COVID-19 patients (Ps) was tested against recombinant full-length SARS-CoV-2 N protein to determine specificity and the optimal serum/plasma dilution. (B) The IgG response of individual HCs and Ps were also assayed to determine the frequency of non-specific binding in HCs.

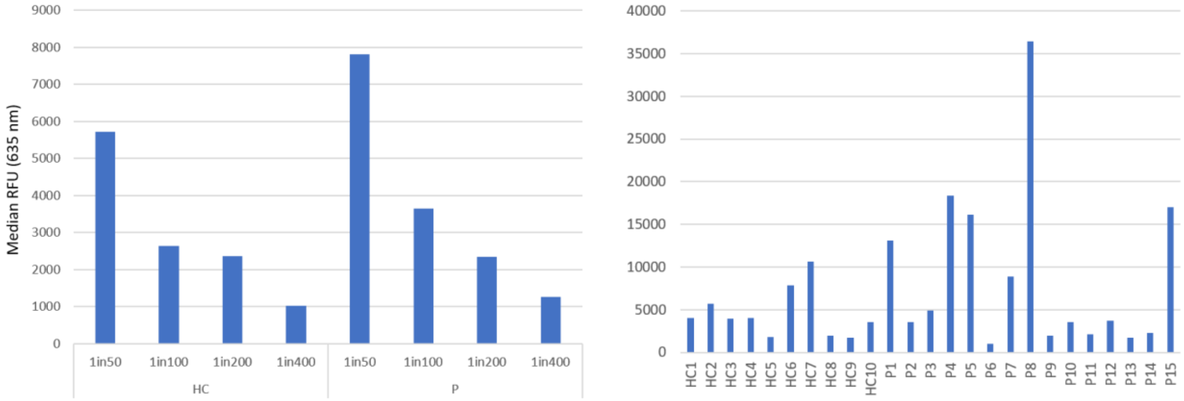

Figure S9. Antibody response to peptide 4. The IgG response of pooled healthy controls (HCs) and convalescent COVID-19 patients (Ps) was assessed against peptide 4. (A) A serial dilution (1:50, 1:100, 1:200 and 1:400) of pooled healthy controls (HCs) and convalescent COVID-19 patients (Ps) was tested against recombinant full-length SARS-CoV-2 N protein to determine specificity and the optimal serum/plasma dilution. (B) The IgG response of individual HCs and Ps were also assayed to determine the frequency of non-specific binding in HCs.

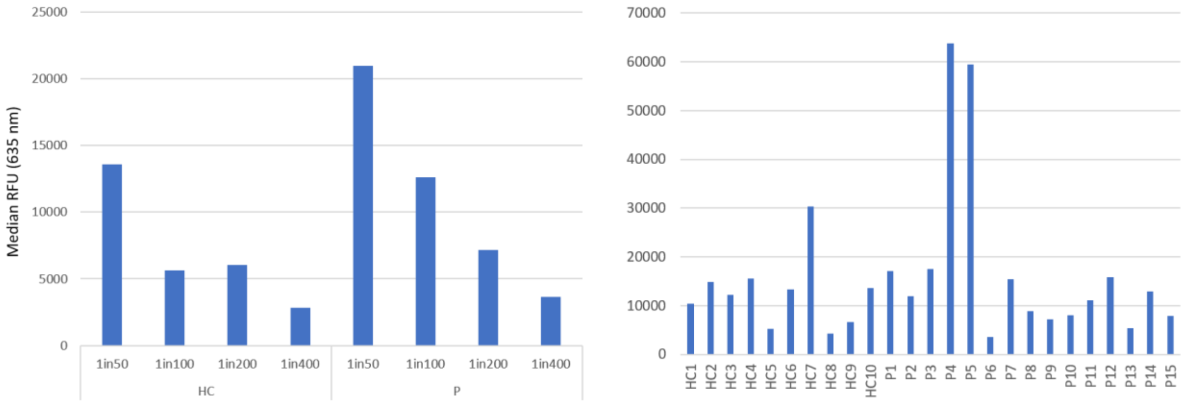

Figure S10. Antibody response to peptide 5. The IgG response of pooled healthy controls (HCs) and convalescent COVID-19 patients (Ps) was assessed against peptide 5. (A) A serial dilution (1:50, 1:100, 1:200 and 1:400) of pooled healthy controls (HCs) and convalescent COVID-19 patients (Ps) was tested against recombinant full-length SARS-CoV-2 N protein to determine specificity and the optimal serum/plasma dilution. (B) The IgG response of individual HCs and Ps were also assayed to determine the frequency of non-specific binding in HCs.

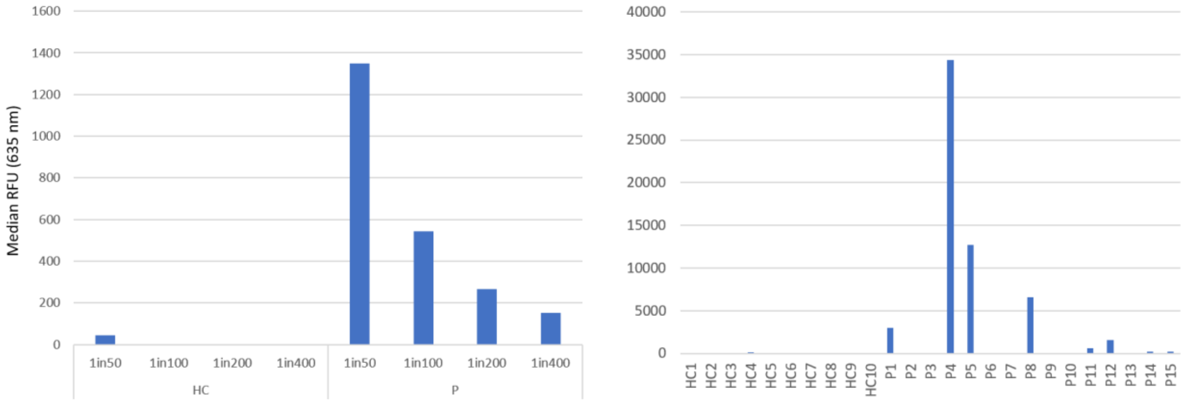

Figure S11. Antibody response to peptide 6. The IgG response of pooled healthy controls (HCs) and convalescent COVID-19 patients (Ps) was assessed against peptide 6. (A) A serial dilution (1:50, 1:100, 1:200 and 1:400) of pooled healthy controls (HCs) and convalescent COVID-19 patients (Ps) was tested against recombinant full-length SARS-CoV-2 N protein to determine specificity and the optimal serum/plasma dilution. (B) The IgG response of individual HCs and Ps were also assayed to determine the frequency of non-specific binding in HCs.

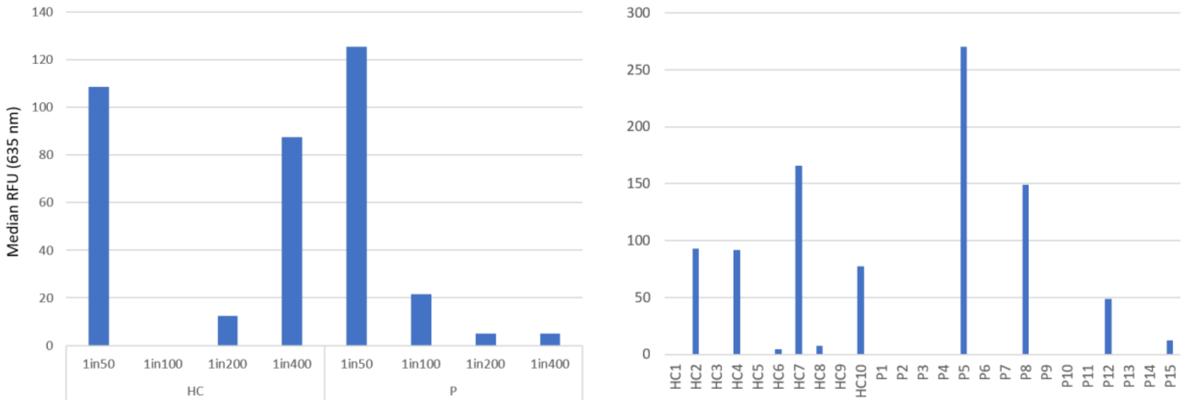

Figure S12. Antibody response to peptide 7. The IgG response of pooled healthy controls (HCs) and convalescent COVID-19 patients (Ps) was assessed against peptide 7. (A) A serial dilution (1:50, 1:100, 1:200 and 1:400) of pooled healthy controls (HCs) and convalescent COVID-19 patients (Ps) was tested against recombinant full-length SARS-CoV-2 N protein to determine specificity and the optimal serum/plasma dilution. (B) The IgG response of individual HCs and Ps were also assayed to determine the frequency of non-specific binding in HCs.

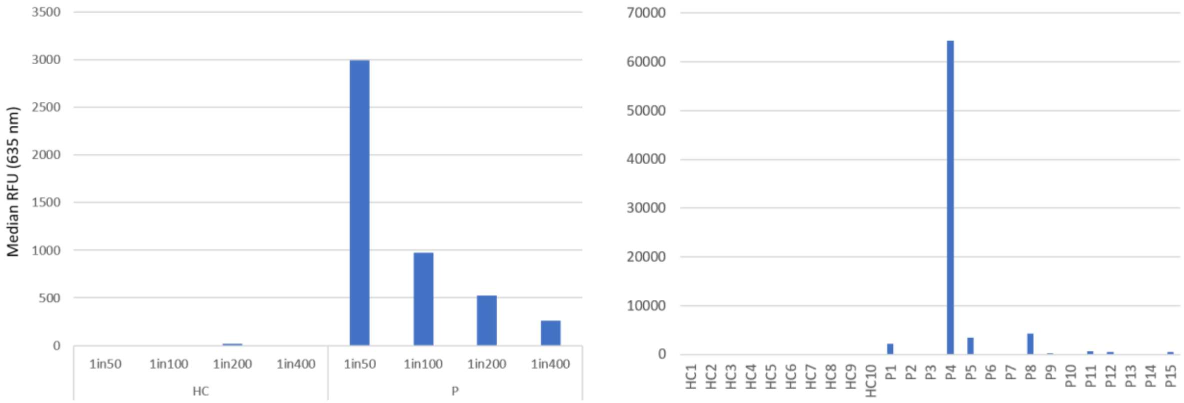

Figure S13. Antibody response to peptide 8. The IgG response of pooled healthy controls (HCs) and convalescent COVID-19 patients (Ps) was assessed against peptide 8. (A) A serial dilution (1:50, 1:100, 1:200 and 1:400) of pooled healthy controls (HCs) and convalescent COVID-19 patients (Ps) was tested against recombinant full-length SARS-CoV-2 N protein to determine specificity and the optimal serum/plasma dilution. (B) The IgG response of individual HCs and Ps were also assayed to determine the frequency of non-specific binding in HCs**.**

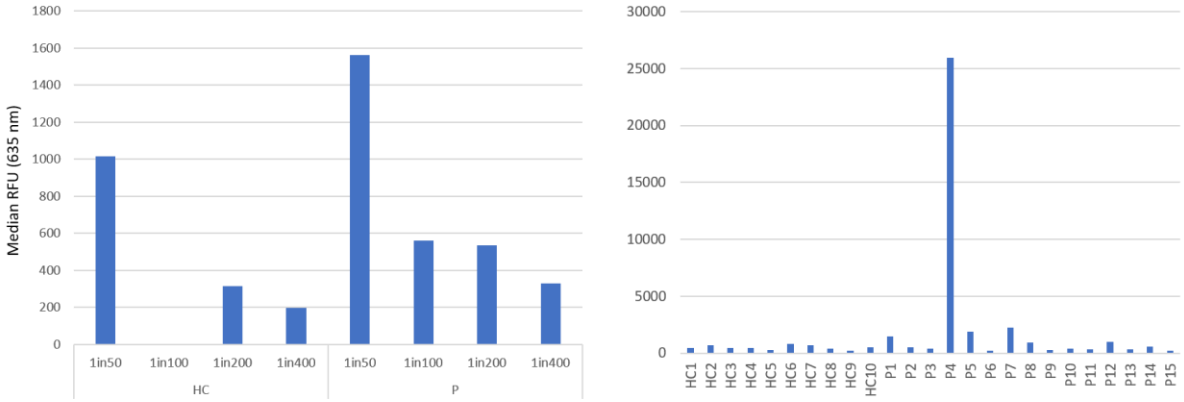

Figure S14. Antibody response to peptide 9. The IgG response of pooled healthy controls (HCs) and convalescent COVID-19 patients (Ps) was assessed against peptide 9. (A) A serial dilution (1:50, 1:100, 1:200 and 1:400) of pooled healthy controls (HCs) and convalescent COVID-19 patients (Ps) was tested against recombinant full-length SARS-CoV-2 N protein to determine specificity and the optimal serum/plasma dilution. (B) The IgG response of individual HCs and Ps were also assayed to determine the frequency of non-specific binding in HCs**.**

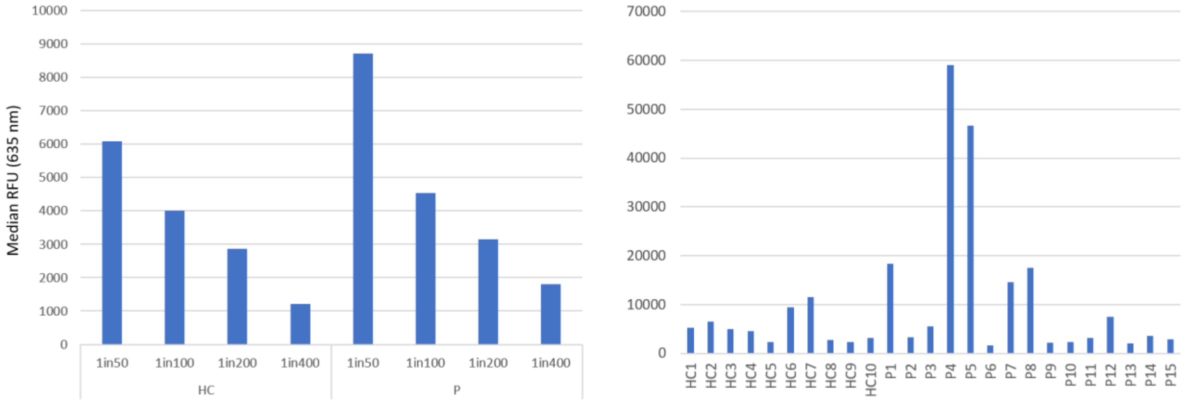

Figure S15. Antibody response to peptide 10. The IgG response of pooled healthy controls (HCs) and convalescent COVID-19 patients (Ps) was assessed against peptide 10. (A) A serial dilution (1:50, 1:100, 1:200 and 1:400) of pooled healthy controls (HCs) and convalescent COVID-19 patients (Ps) was tested against recombinant full-length SARS-CoV-2 N protein to determine specificity and the optimal serum/plasma dilution. (B) The IgG response of individual HCs and Ps were also assayed to determine the frequency of non-specific binding in HCs.

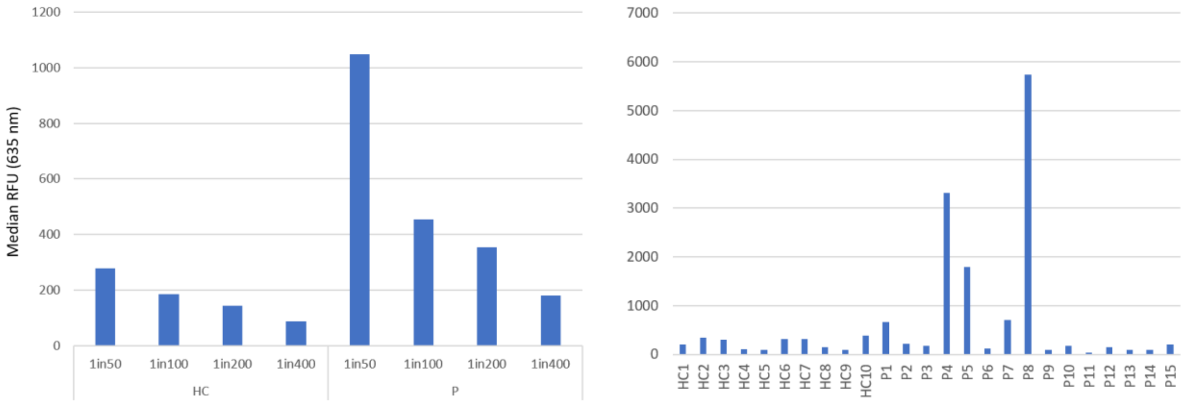

Figure S16. Antibody response to peptide 11. The IgG response of pooled healthy controls (HCs) and convalescent COVID-19 patients (Ps) was assessed against peptide 11. (A) A serial dilution (1:50, 1:100, 1:200 and 1:400) of pooled healthy controls (HCs) and convalescent COVID-19 patients (Ps) was tested against recombinant full-length SARS-CoV-2 N protein to determine specificity and the optimal serum/plasma dilution. (B) The IgG response of individual HCs and Ps were also assayed to determine the frequency of non-specific binding in HCs.

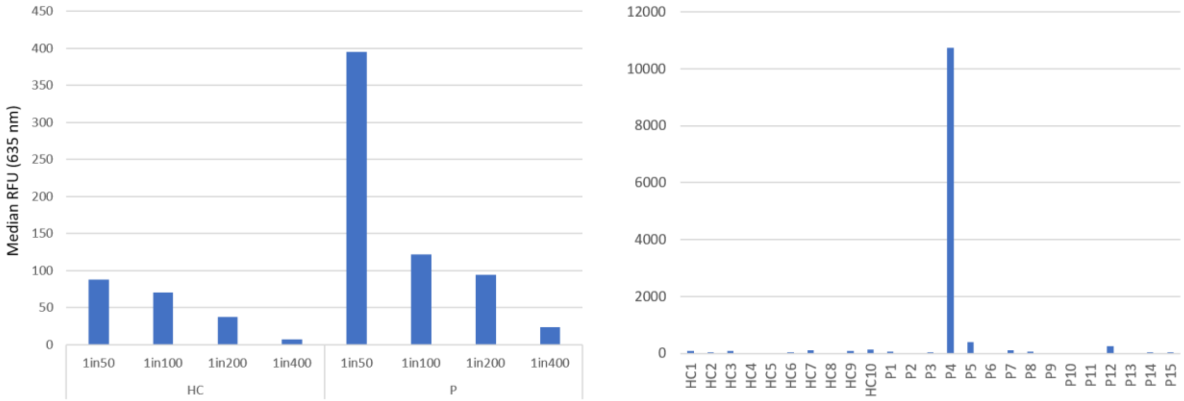

Figure S17. Antibody response to peptide 12. The IgG response of pooled healthy controls (HCs) and convalescent COVID-19 patients (Ps) was assessed against peptide 12. (A) A serial dilution (1:50, 1:100, 1:200 and 1:400) of pooled healthy controls (HCs) and convalescent COVID-19 patients (Ps) was tested against recombinant full-length SARS-CoV-2 N protein to determine specificity and the optimal serum/plasma dilution. (B) The IgG response of individual HCs and Ps were also assayed to determine the frequency of non-specific binding in HCs.

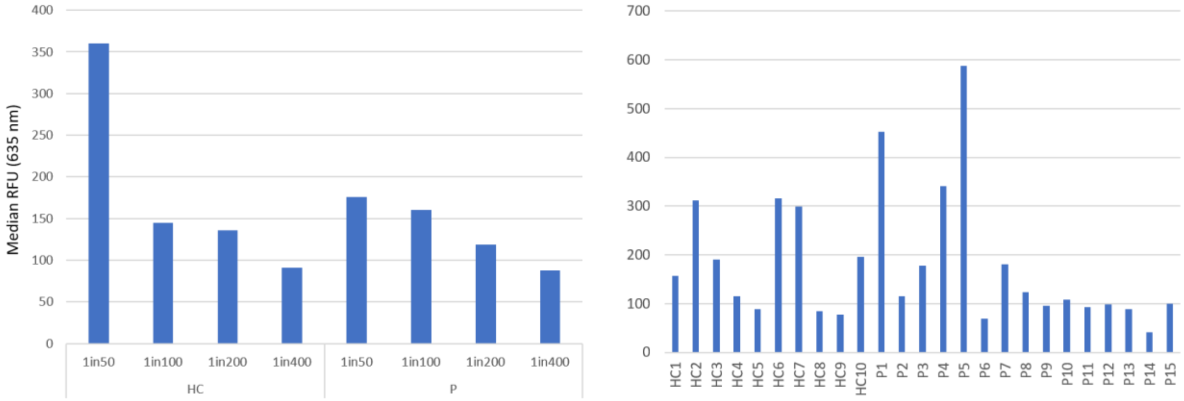

Figure S18. Antibody response to peptide 13. The IgG response of pooled healthy controls (HCs) and convalescent COVID-19 patients (Ps) was assessed against peptide 13. (A) A serial dilution (1:50, 1:100, 1:200 and 1:400) of pooled healthy controls (HCs) and convalescent COVID-19 patients (Ps) was tested against recombinant full-length SARS-CoV-2 N protein to determine specificity and the optimal serum/plasma dilution. (B) The IgG response of individual HCs and Ps were also assayed to determine the frequency of non-specific binding in HCs.

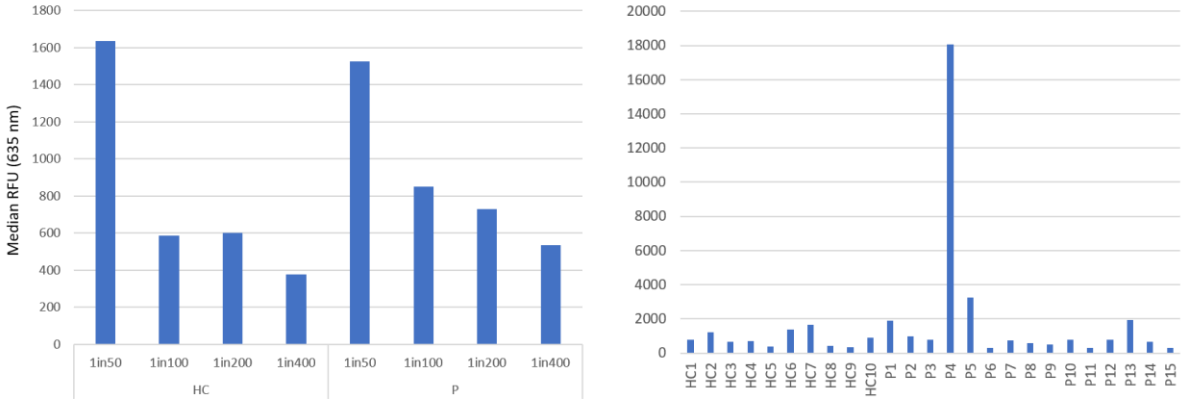

Figure S19. Antibody response to peptide 14. The IgG response of pooled healthy controls (HCs) and convalescent COVID-19 patients (Ps) was assessed against peptide 14. (A) A serial dilution (1:50, 1:100, 1:200 and 1:400) of pooled healthy controls (HCs) and convalescent COVID-19 patients (Ps) was tested against recombinant full-length SARS-CoV-2 N protein to determine specificity and the optimal serum/plasma dilution. (B) The IgG response of individual HCs and Ps were also assayed to determine the frequency of non-specific binding in HCs.

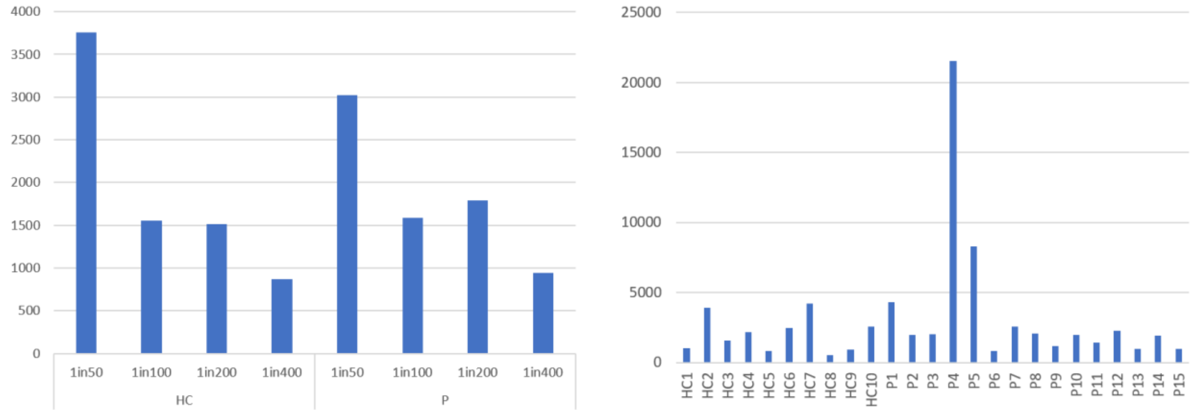

Figure S20. Antibody response to peptide 15. The IgG response of pooled healthy controls (HCs) and convalescent COVID-19 patients (Ps) was assessed against peptide 15. (A) A serial dilution (1:50, 1:100, 1:200 and 1:400) of pooled healthy controls (HCs) and convalescent COVID-19 patients (Ps) was tested against recombinant full-length SARS-CoV-2 N protein to determine specificity and the optimal serum/plasma dilution. (B) The IgG response of individual HCs and Ps were also assayed to determine the frequency of non-specific binding in HCs.

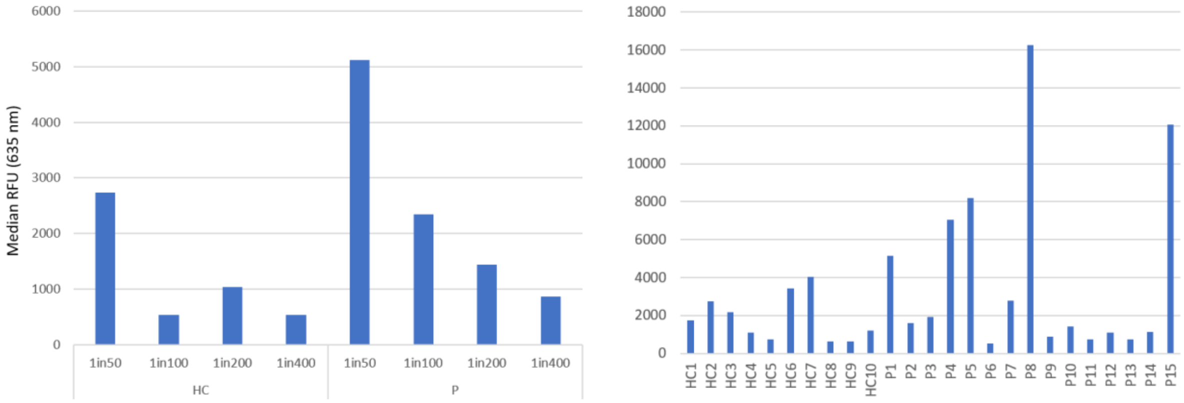

Figure S21. Antibody response to peptide 16. The IgG response of pooled healthy controls (HCs) and convalescent COVID-19 patients (Ps) was assessed against peptide 16. (A) A serial dilution (1:50, 1:100, 1:200 and 1:400) of pooled healthy controls (HCs) and convalescent COVID-19 patients (Ps) was tested against recombinant full-length SARS-CoV-2 N protein to determine specificity and the optimal serum/plasma dilution. (B) The IgG response of individual HCs and Ps were also assayed to determine the frequency of non-specific binding in HCs.

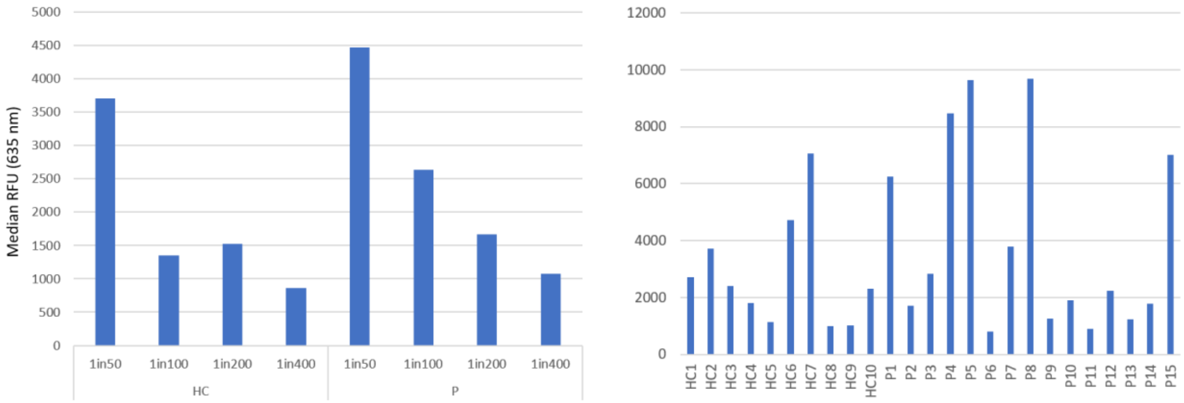

Figure S22. Antibody response to peptide 17. The IgG response of pooled healthy controls (HCs) and convalescent COVID-19 patients (Ps) was assessed against peptide 17. (A) A serial dilution (1:50, 1:100, 1:200 and 1:400) of pooled healthy controls (HCs) and convalescent COVID-19 patients (Ps) was tested against recombinant full-length SARS-CoV-2 N protein to determine specificity and the optimal serum/plasma dilution. (B) The IgG response of individual HCs and Ps were also assayed to determine the frequency of non-specific binding in HCs.

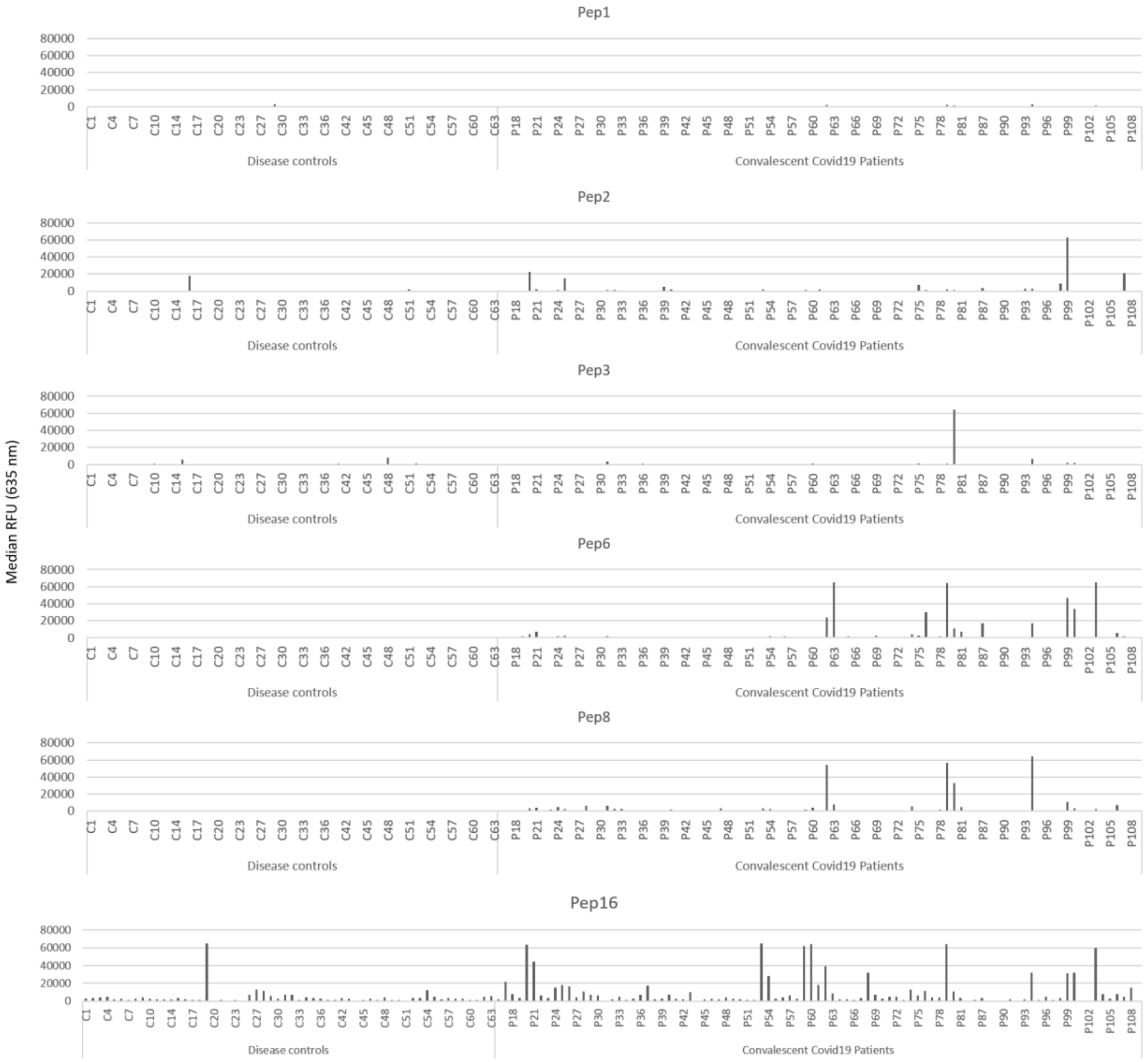

Figure S23. IgG response to 6 peptides of the SARS-CoV-2 N protein. Peptides 1, 2, 3, 6, 8 and 16 from the SARS-CoV-2 N protein were fabricated on the microarray surface. The IgG response of 58 colorectal cancer patients (Cs) and 91 convalescent COVID-19 patients (Ps) were assessed on the microarray to determine which epitope coverage and improve platform sensitivity and specificity.

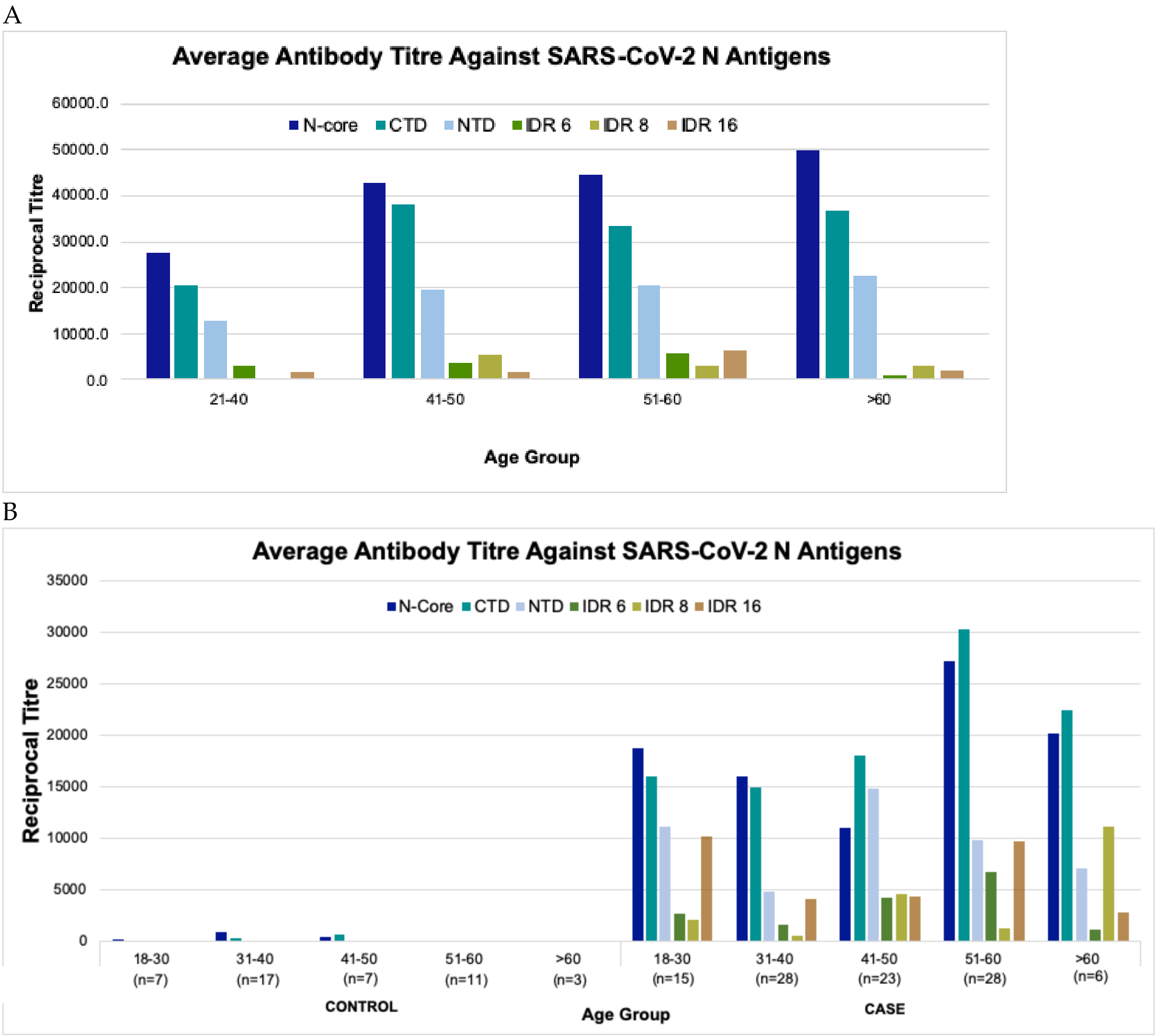

Figure S24. Histogram displaying the antibody titres across age groups against different epitopes of the SARS-CoV-2 N protein. (A) Validation Cohort 2, (B) Multi-ethnic Cohort 3 (all PCR+ patients, both mild and severe).

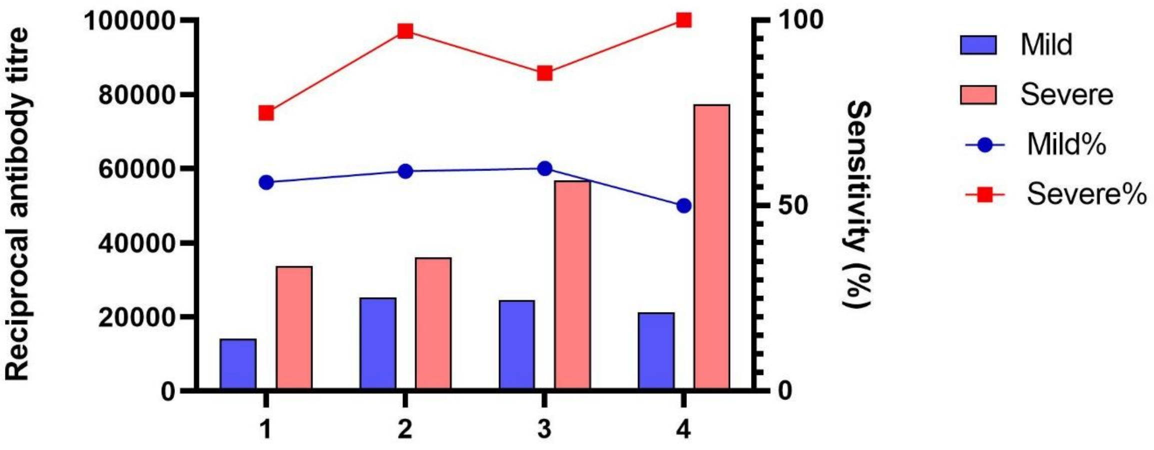

Figure S25. Average antibody titre and sensitivity for Cohort 3 patients with mild and severe disease (1 = 0-7 days, 2 = 8-14 days, 3 = 15-21, 4 = >21).
